# A Cohort Study on Cerebrovascular Disease in Middle-aged and Elderly Population in Rural Areas in Jiangxi Province, China

**DOI:** 10.2188/jea.13.149

**Published:** 2007-11-30

**Authors:** Dongmei Qiu, Jiamo Mei, Takeo Tanihata, Katsuhiko Kawaminami, Masumi Minowa

**Affiliations:** 1 Department of Epidemiology and Environmental Health, Juntendo University School of Medicine.; 2 Jiangxi Center for Disease Control and Prevention.; 3 Department of Epidemiology, National Institute of Public Health.

**Keywords:** cohort study, cerebrovascular disease, mortality rate, hazard ratio, rural area, China

## Abstract

To clarify the risk factors of CVD deaths in rural areas in Jiangxi Province, China, a cohort study was carried out from September 1, 1994 through December 31, 2000 involving 50,252 participants aged 40 years or older in 4 counties. Among the 3,429 deaths, 671 cases (398 males and 273 females) died of CVD. In addition, excluding 183 cases with a previous history of CVD, 632 CVD deaths out of 50,069 subjects were analyzed using Cox proportional hazard models. The multivariate hazard ratio (HR) for CVD mortality significantly increased in parallel with age, blood pressure and degree of liking for salty foods (p for trend<0.01). The multivariate HR for CVD mortality of ex-drinkers was 1.55 (95%CI: 1.04, 2.31) compared with non-drinkers. The multivariate HR for CVD mortality of subjects who ate meat once or twice per month was 0.75 (95%CI: 0.62, 0.91) compared with those who never ate meat or seldom. There was no significant relationship between smoking and CVD mortality. Our results indicated that the main risk factors for CVD mortality were advancing age, high-normal blood pressure and hypertension. The risk in these areas was lower in subjects who disliked salty foods and those who ate meat once or twice per month.

Disease patterns have been showing a notable change in China following the rapid socioeconomic changes caused by economic reforms. Chronic non-communicable diseases have superseded communicable diseases as the principal cause of death in China.^1^ This change has occurred not only in urban areas but also in rural areas. In both the areas, cerebrovascular disease (CVD) has become the second largest cause of death in China. The mortality of CVD increased from 39.1/100,000 in 1957 to 149.5/100.000 in 1998 (according to data of the Ministry of Health, People’s Republic of China). Moreover, it increased more rapidly in rural areas than in urban areas.^1^ It is reported that more than 1 million people die of CVD in a year in China.^2^ The high mortality and high disability rates of CVD have not only affected the health and quality of life of the victims, but also caused heavy economic and mental burdens for families and the country. Furthermore, the burden of CVD is likely to increase substantially in the future because of the aging population and the changing of lifestyles. Thus, it is necessary to take effective measures against CVD as soon as possible. Until now there have been no sufficient epidemiologic studies carried out in rural areas in China.^3^^,^^4^ The present study was conducted to explore the risk factors associated with CVD deaths in the rural areas in Jiangxi Province, China. We hope that our results may provide public health information on the prevention and management of CVD, as well as suggestions for relieving the burden caused by CVD.

## METHODS

The investigation was performed with persons who were 40 years or older who lived in the study areas, Sixi, Lixi, Luoping, Putian, Luxi, Shinao, Meizhuang, and Ertang Townships in Shanggao, Wuning, Jinxian, and Gaoan Counties in Jiangxi Province, China. Inpatients were excluded from the study. Jiangxi Province, with a total population of about 42 million, is located in the middle southwest of China, along the middle lower reaches of the Yangtze River. Among the population in the province, approximately 60% are farmers, 25% work in industries, such as excavation and manufacturing, and 15% are others (Jiangxi CDC, personal communication). In the investigating areas, more than 90% are farmers, and rice is the main crop. In 1993, the birth rate and mortality rate in this province were 20.3‰ and 6.9‰, respectively, while the birth rate and mortality rate in the whole country were 18.1‰ and 6.6‰, respectively. Average rural household per capita net income in Jiangxi Province is in the middle of 31 metropolises and provinces in China.^5^ The climate in the province is relatively warm and moist; the average temperature is 16.3°C∼19.5°C and the annual precipitation of rainfall is 1,351∼1,934 mm.^6^

Between September 1, 1994 and June 30, 1996, a door-to-door baseline survey about lifestyle and health status was performed with 50,252 subjects (93.3% of eligible subjects) by an investigating team using a structured questionnaire.

The institutions involved in the survey, ranging from basic to high level facilities included village clinics, township and county hospitals, Centers for Disease Control and Prevention (CDC) from county to provincial level. Physicians who belonged to county CDC or lower levels were trained by those of Jiangxi CDC for one week before conducting the baseline survey. About the 300 physicians participated in this survey. All the interviews were supervised by the physicians from the Jiangxi CDC, and they were also responsible for checking the finished questionnaires.

The surveyed items were sex, age, frequency of food intake (meat, egg, fresh fish, Chinese pickles, tofu, green-yellow vegetables, light-colored vegetables, milk, confectionery, and fruit), liking for fatty foods and salty foods, cigarette smoking, alcohol drinking, occupation, living environment, life situation, and past histories of CVD, heart disease, hypertension and malignant neoplasm. Concerning the diseases, in addition to confirmation by the medical records preserved by the subjects themselves, they were also diagnosed by the medical examinations made by physicians of the county and township hospitals in the subjects’ homes. Further clinical examinations were carried out in the township hospitals or the county hospitals if necessary. Other information collected at the baseline survey included the measurement of height, weight, and blood pressure. According to the criteria of China,^7^ body mass index (BMI) was classified into three groups as; BMI<18.5, 18.5 ≦ BMI ≦ 23.9 and BMI ≧ 24.0. The value of the blood pressure was the mean of 3 time measurements. When systolic blood pressure (SBP)<130 mmHg and diastolic blood pressure (DBP)<85 mmHg, it was classified as normal; when 130 ≦ SBP ≦ 139 mmHg and/or 85 ≦ DPB ≦ 89 mmHg, it was classified as “high-normal”; when SBP ≧ 140 mmHg and/or DPB ≧ 90 mmHg, it was classified as hypertension according to the reference of WHO/ ISH.^8^ Subjects who had histories of hypertension were regarded as cases of hypertension.

As of December 31, 2000, among the 50,252 cohort subjects, only 225 subjects had moved away from the investigating areas (and therefore had been lost to follow-up). Regarding mortality data, follow-up was essentially complete (follow-up rate was 99.6%).

A follow-up survey (including date of death and causes of death) of the subjects was monitored regularly by means of a follow-up report. Village physicians who lived in the same village as cohort subjects filled out follow-up reports and submitted them to the township hospitals every 10 days. After receiving a follow-up report, a physician from the township hospital immediately visited the home of the dead person, and after the township hospital physician confirmed the diagnosis, he or she submitted a report to the county CDC once per month. A physician from the county CDC visited the home of any dead person who could not be confirmed by the township hospital. The county CDC submitted the reports to the Jiangxi CDC quarterly during the year. We obtained the data from the Jiangxi CDC annually. Causes of death were determined mainly from death certificates, supplemented if necessary, by medical records and by asking family members or the village physicians. Survivors and subjects who had moved away from the investigating areas were confirmed by using registration of residents in local police stations annually.

The investigating team not only explained the purpose, methods, procedure, the meaning and advantage of this survey, but also emphasized voluntary nature of participation in the survey to the residents before interview. All the interviews were performed only with residents who were willing to participate in this study.

Each subject accumulated follow-up time beginning at the baseline and ending at the date of death or other relevant endpoint (death from causes other than CVD, or December 31, 2000, whichever came first). In the death cases, causes of deaths were coded according to the basic tabulation list of the International Classification of Diseases, Ninth Revision (ICD-9). The specific endpoints of this study were deaths from CVD (ICD-9:29), which were cerebral hemorrhage (ICD-9: 291), cerebral infarction (ICD-9: 292), subarachnoid hemorrhage (ICD-9: 290) and other CVD which could not be clearly diagnosed (ICD-9: 293-294).

We selected confounding factors on the principle of p < 0.20 according to Wald’s test and took into account the importance of variables. We used Cox proportional hazards models to estimate the risk of mortality (cumulative mortality) by CVD, adjusting for sex, age and other covariates. Selected confounders were sex (males and females), age group (40-49, 50-59, 60-69, 70-79, and 80+ years), area (Wuning county, Shanggao county, Jinxian county, and Gaoan county), cigarette smoking status (non-smoker, ex-smoker, and current smoker), alcohol drinking status (nondrinker, ex-drinker, and current drinker), blood pressure (normal, high-normal, and hypertension), BMI (<18.5, 18.5-23.9, and ≧ 24.0 kg/m^2^), marital status (married, never married, divorced, and widowed), fatty foods (dislike, normal, and like), salty foods (dislike, normal, and like), frequency of Chinese pickles intake (never or seldom, once or twice per month, and more than once per week), frequency of meat intake (never or seldom, once or twice per month, and more than once per week), sleeping hours per day (6 hours or less, 7 to 8 hours per day, and 9 hours or more). The 95% confidence interval (CI) was calculated for each hazard ratio (HR). We excluded 183 cases who had suffered from CVD before the baseline date when analyzing the causes of death by CVD. Statistical testing for p for trend of CVD mortality associated with various variables was based on ordinal categories using Cox proportional hazard models. Data were analyzed with SPSS^®^10.0 for Windows.

## RESULTS

For 50,252 subjects (25,338 males and 24,914 females), the mean age was 55.3 years old. The number of current smokers was far greater than that of ex-smokers. A similar difference was observed in regard to current drinkers and ex-drinkers. The prevalence of hypertension was 20.4%. Mean BMI was 20.1 kg/m^2^ (For details, see Table 1).

**Table 1.** Distributions of selected characteristics of cohort participants at baseline, Jiangxi Province, China, between 1994 and 1996.

	Males	Females	Total
No. of subjects	25338	24914	50252

Age group (%)			
40-49 years	41.6	40.6	41.1
50-59	24.4	21.6	23.0
60-69	20.6	20.9	20.8
70-79	11.3	13.3	12.3
80+	2.1	3.5	2.8

Mean age (years) ± SD	54.8 ± 11.4	55.8 ± 12.2	55.3 ± 11.8

Area (%)			
Wuning	40.7	38.6	39.7
Shanggao	19.9	19.5	19.7
Gaoan	18.6	20.5	19.6
Jinxian	20.7	21.4	21.0

Cigarette smoking status (%)			
Non-smoker	21.5	93.4	57.1
Ex-smoker	5.4	0.6	3.0
Current smoker	73.1	6.1	39.9

Alcohol drinking status (%)			
Non- drinker	36.7	78.1	57.2
Ex-drinker	2.6	1.4	2.0
Current drinker	60.6	20.5	40.8

Blood pressure * (%)			
Normal ^#^	62.9	66.7	64.8
High-normal ^##^	16.7	13.0	14.8
Hypertension ^###^	20.4	20.3	20.4

Mean body mass index (kg/m^2^) ± SD**	20.3 ± 2.3	20.0 ± 2.7	20.1 ± 2.5

* Categories followed the criteria of WHO. SBP: systolic blood pressure (mmHg); DBP: diastolic blood pressure (mmHg).

^#^ Normal (SBP<130 and DBP<85).

^##^ High-normal (130 ≦ SBP ≦ 139 and/or 85 ≦ DBP ≦ 89).

^###^ Hypertension (SBP ≧ 140 and/or DBP ≧ 90 and those who had histories of hypertension).

** SD: Standard deviation.

By December 31, 2000, except for 183 cases that had a history of CVD, 50,069 cohort participants (25,226 males and 24,843 females) had contributed 257,568 person-years of follow-up. During about 6 years of follow-up, 3,358 (2,018 males and 1,340 females) deaths occurred, 46,486 (23,074 males and 23, 412 females) persons survived, and 225 (134 males and 91 females) persons had moved (Table 2). Among the 3,358 deaths, 632 (377 males and 255 females) cases died of CVD, of which 432 and 76 were cerebral hemorrhage and cerebral infarction, respectively (Table 3).

**Table 2.** Distributions of followed-up results (%) (from September 1994* through December 2000).

	Males		Females		Total	
Died	2018	( 8.0)	1340	( 5.4)	3358	( 6.7)
Survived	23074	( 91.5)	23412	( 94.2)	46486	( 92.8)
Removed	134	( 0.5)	91	( 0.4)	225	( 0.4)
Total	25226	(100.0)	24843	(100.0)	50069	(100.0)

* The entry date for each investigated area was different from 1994 to 1996.

Note: The cases who had a history of cerebrovascular disease were excluded.

**Table 3.** Number of deaths by cerebrovascular disease and other diseases * (%).

	Males		Females		Total	
Cerebrovascular disease *	377	( 18.7)	255	( 19.0)	632	( 18.8)
Cerebral hemorrhage **	257	( 12.7)	175	( 13.1)	432	( 12.9)
Cerebral infarction ^#^	45	( 2.2)	31	( 2.3)	76	( 2.3)
Subarachnoid hemorrhage ^##^	22	( 1.1)	12	( 0.9)	34	( 1.0)
Uncertain cerebrovascular diseases type ^$^	53	( 2.6)	37	( 2.8)	90	( 2.7)
Other causes	1641	( 81.3)	1085	( 81.0)	2726	( 81.2)
Total	2018	(100.0)	1340	(100.0)	3358	(100.0)

* The category of the basic tabulation list of ICD-9 (International Classification of Diseases, Ninth Revision) is 29.

** The subcategory is 291.

^#^ The subcategory is 292.

^##^ The subcategory is 290.

^$^ The subcategories are 293-294.

Note: The cases who had a history of cerebrovascular disease were excluded.

As shown in Table 4, the HR of CVD mortality rate increased rapidly with advancing age (p for trend < 0.01). Adjustment for sex, area and other covariates listed in Table 4 did not affect the positive increase tendency (p for trend<0.01). The HRs and multivariate HRs of CVD mortality rates approximately doubled every 10 years from the 40-49 age groups to 80+ age groups. The HR of CVD mortality rate increased about 29-fold when the oldest group (80+ age group) and the 40-49 age groups were compared. The multivariate HR of CVD mortalities rate in the 80+ age group was 19.85 compared with the 40-49 age group.

**Table 4.** Hazard ratios of cerebrovascular disease mortality.*

	Person-years	No. of deaths	HR^/^	95%CI **	Multivariate HR^//^	95%CI **
(Age group)						
40-49 years	108556	59	1.00		1.00	
50-59	60423	77	2.32	( 1.65 - 3.26)	2.14	( 1.52 - 3.01)
60-69	53203	177	6.18	( 4.61 - 8.30)	5.13	( 3.79 - 6.94)
70-79	29444	234	15.16	(11.39 - 20.17)	11.38	( 8.34 - 15.54)
80+	5942	85	28.78	(20.63 - 40.16)	19.85	(13.65 - 28.87)
p for trend			<0.01		<0.01	

(Cigarette smoking status)						
Non-smoker	146469	320	1.00		1.00	
Ex-smoker	7034	43	1.52	( 1.08 - 2.15)	1.40	( 0.98 - 2.00)
Current smoker	102939	264	1.12	( 0.90 - 1.38)	1.08	( 0.87 - 1.34)
p for trend			0.41		0.59	

(Alcohol drinking status)						
Non- drinker	147088	341	1.00		1.00	
Ex-drinker	4768	28	1.69	( 1.14 - 2.49)	1.55	( 1.04 - 2.31)
Current drinker	105529	261	1.18	( 0.99 - 1.40)	1.12	( 0.93 - 1.34)
p for trend			0.06		0.23	

(Blood pressure)^¶^						
Normal ^#^	169071	239	1.00		1.00	
High-normal ^##^	37862	102	1.40	( 1.11 - 1.77)	1.38	( 1.09 - 1.74)
Hypertension ^###^	50636	291	2.12	( 1.78 - 2.54)	2.06	( 1.72 - 2.47)
p for trend			<0.01		<0.01	

(Body mass index)^7^						
<18.5	64355	222	1.00		1.00	
18.5-23.9	177928	385	1.13	( 0.95 - 1.34)	1.12	( 0.94 - 1.33)
≧24.0	15285	25	1.14	( 0.75 - 1.73)	1.03	( 0.68 - 1.58)
p for trend			0.18		0.33	

(Marital status)						
Married	214233	399	1.00		1.00	
Never married	2870	7	1.23	( 0.58 - 2.61)	1.25	( 0.59 - 2.65)
Divorced	2617	6	1.01	( 0.45 - 2.26)	0.95	( 0.42 - 2.13)
Widowed	37849	220	1.22	( 1.01 - 1.47)	1.16	( 0.96 - 1.41)
p for trend			-		-	

(Fatty foods)						
Dislike	6401	14	1.00		1.00	
Normal	93626	241	1.28	( 0.75 - 2.20)	1.24	( 0.72 - 2.15)
Like	157542	377	1.37	( 0.80 - 2.33)	1.33	( 0.78 - 2.29)
p for trend			0.23		0.23	

(Salty foods)						
Dislike	60659	112	1.00		1.00	
Normal	165613	433	1.41	( 1.15 - 1.74)	1.40	( 1.13 - 1.73)
Like	31296	87	1.53	( 1.15 - 2.02)	1.46	( 1.10 - 1.95)
p for trend			<0.01		<0.01	

(Frequency of Chinese pickles intake)						
Never or seldom	159945	412	1.00		1.00	
Once or twice per month	48500	116	0.92	( 0.75 - 1.13)	0.91	( 0.74 - 1.13)
More than once per week	49123	104	0.86	( 0.69 - 1.06)	0.79	( 0.63 - 0.98)
p for trend			0.14		0.03	

(Frequency of meat intake)						
Never or seldom	52002	165	1.00		1.00	
Once or twice per month	142481	322	0.77	( 0.64 - 0.93)	0.75	( 0.62 - 0.91)
More than once per week	63085	145	0.83	( 0.66 - 1.04)	0.84	( 0.66 - 1.06)
p for trend			0.10		0.13	

Sleeping hours per day						
6 hours or less	21465	97	1.00		1.00	
7 to 8 hours	180847	376	0.78	( 0.62 - 0.98)	0.86	( 0.68 - 1.09)
9 hours or more	55257	159	0.96	( 0.74 - 1.23)	1.01	( 0.78 - 1.31)
p for trend			0.81		0.65	

* Missing values and the cases who had a history of cerebrovascular disease were excluded.

^/^ HR, hazard ratio adjusted for sex and category of age (40-49, 50-59, 60-69, 70-79, and 80+ years).

^//^ Multivariate HR, hazard ratio adjusted for sex, different areas (Wuning, Shanggao, Gaoan and Jinxian County) and the factors listed in this table.

** CI, confidence interval.

^¶^ Category was according to criterion of WHO. SBP: systolic blood pressure (mmHg); DBP: diastolic blood pressure (mmHg).

^#^ Normal (SBP<130 and DBP<85).

^##^ High-normal (130 ≦ SBP ≦ 139 and/or 85 ≦ DBP ≦ 89).

^###^ Hypertension (SBP ≧ 140 and/or DBP ≧ 90 and those who had histories of hypertension).

When compared with non-drinkers, ex-drinkers showed a 69% significant increase in HR of CVD mortality rate. The significant increase was also shown in the multivariate analysis (HR=1.55). The HR of CVD mortality rate for current drinkers showed a borderline significant increase when compared with non-drinkers (HR=1.18). However, in the multivariate analysis, this increase was not significant.

The tendency of risk for CVD mortality rate significantly increased with blood pressure (p for trend < 0.01). A similar trend was observed in regard to the multivariate HR (p for trend < 0.01). Compared with subjects with normal blood pressure, the HRs for those with high-normal blood pressure and those with hypertension were 1.40 and 2.12, respectively. Compared with subjects with normal blood pressure, the multivariate HRs for those with high-normal blood pressure and those with hypertension were 1.38 and 2.06, respectively.

The HR of CVD mortality rate showed a significant increasing tendency associated with degree of liking for salty foods (p for trend < 0.01). According to multivariate analysis, a significant increasing tendency was also observed (p for trend<0.01). Compared with subjects who disliked salty foods, the HRs of CVD mortality rates for those who had normal feelings about salty foods and those who liked salty foods were 1.41 and 1.53, respectively. The multivariate HRs of CVD mortality rates for subjects who had normal feelings about salty foods and those who liked salty foods were 1.40 and 1.46, respectively, compared with subjects who disliked salty foods. There was no significant association between Chinese pickles (salty foods) intake and CVD mortality rate in the univariate analysis. However, the HR of CVD mortality rate showed a significant reducing tendency with increasing frequency of Chinese pickles intake in multivariate analysis (p for trend = 0.03). Compared with subjects who never ate Chinese pickles or ate them seldom, the HR of CVD mortality rate for those who ate Chinese pickles more than once per week was less than unity.

The HR of CVD mortality rate decreased in subjects who ate meat (including chicken, pork, and beef) once or twice per month and those who ate meat more than once per week (subjects who never ate meat or ate it seldom were the reference). The HR of CVD was significant only in subjects who ate meat once or twice per month (HR =0.77). A similar result was found in using the multivariate analysis (HR=0.75); however, there was no significant association between degree of liking for fatty foods and CVD mortality rate. Furthermore, no significant association was found between any category of liking for fatty foods and CVD mortality rate.

Regarding both crude and adjusted HRs, we found that there was no significant association between BMI and CVD mortality rate. Although ex-smokers showed a significant increase in HR for CVD mortality rate when compared with non-smokers, this significant increase was not observed in the multivariate analysis. Significant increase in HR for CVD mortality rate was observed in widowers (currently married subjects were reference); significant decrease was observed in subjects who slept 7 to 8 hours per day (subjects who slept 6 hours or less were reference). However, both of these significant differences disappeared in the multivariate analysis.

## DISCUSSION

As shown in Table 1, the current smoking rate among males (73.1%) in our study was higher than that in China nationwide (66.8%),^9^ and among rural males in Beijing (70.7%);^10^ whereas the current smoking rate among females (6.1%) was lower than that in China nationwide (7.4%).^9^ Current alcohol drinking rates, both among males (60.6%) and females (20.5%) were higher in our study than those in China nationwide (males: 50.4%, females: 5.3%).^9^ The high smoking rate and drinking rate suggested that this subject population might be insufficiently educated about life style for health. The prevalence of hypertension among males (20.4%) and females (20.3%) in our study was lower than those in rural areas in Beijing (males: 21.9%, females: 22.6%; SBP ≧ 160 mmHg and/or DBP ≧ 95 mmHg and normotensives who had taken hypotensive medicine within 2 weeks) respectively.^10^ The mean BMI of males and females in our study (males: 20.3 kg/m^2^; females: 20.0 kg/m^2^) were lower than that in males (23.9 kg/m^2^) and females (24.6 kg/m^2^) in rural areas in Beijing, respectively.^10^ These low prevalences of hypertension and mean BMI may be partly explained by insufficient nutrition (for example, low animal protein) due to low economic state. Based on Table 2 and Table 3, the age-adjusted death rates (standard population was the 1990 Population Census of China, by direct method) of all cause and CVD death rate were calculated. The results (1,305 for all causes of death and 254 for CVD deaths per 100,000 person-years) were lower than those of the same age population in China (1,347/100,000 and 281/100,000), respectively.^11^ Using the same standard population, we also calculated the age-adjusted death rate of CVD of the same age population in Japan in 1970, when Japanese CVD mortality began to decrease. This Japanese age-adjusted death rate for CVD (520/100,000)^12^ was much higher than that in our study. Therefore, we consider that CVD mortality in the investigated areas is likely to increase in the future, following a similar increasing tendency to that of Japan before the 1970s.

When we compared the classification for death causes of CVD in our study with that in Japan around 1960s, we found that more than 50% of deaths were cerebral hemorrhage among CVD deaths both in our study (Table 3) and in Japan.^13^ In analyzing this phenomenon, it should be considered that both in Japan in 1960s (in Japan, the time for introduction of computerized tomography equipment was 1975^14^^)^) and in our study, a large part of the diagnoses of CVD as death cause were not made by computerized tomography, but made according to symptoms and clinical examination only. In the present study, considering the above situation we did not analyze cerebral hemorrhage and cerebral infarction separately.

As shown in Table 4, we found that the risk of CVD mortality rate for middle-aged and elderly population in these areas showed a significant increasing tendency with advancing age, increasing blood pressure and increasing degree of liking for salty foods. The risk in ex-drinkers for CVD mortality rate also showed a significant increase compared with that of non-drinkers. The risk for CVD mortality rate among subjects who ate meat once or twice per month showed a 25% significant reduction compared with those who never ate meat or ate it seldom.

Age was the greatest risk factor for CVD mortality. For every successive 10 years after age 55, the CVD rate more than doubled in both sexes.^15^ Compared with the age 45-54 group, the mortality rate of CVD (only including cerebral hemorrhage and cerebral infarction) in the age 65-74 group increased 19.7 times in rural areas in Tianjin.^4^ This kind of increasing trend was also observed in a study of urban communities in six cities in China.^16^

Hypertension was the other important risk factor for CVD mortality. Hypertension has been independently associated with CVD mortality worldwide.^17^^-^^20^ A history of hypertension was also reported as a primary risk factor of CVD among men in Shanghai.^21^ Elevated blood pressure was a firmly established risk factor for CVD mortality.^19^ Geographic gradient for mortality rate of CVD paralleled the prevalence of hypertension in China.^2^ In a 15-year cohort study conducted in Finland, according to multivariate analysis, it was found that the relative risk of CVD mortalities for people who had hypertension (SBP ≧ 160mmHg and DBP ≧ 95mmHg) were elevated 3.5-fold and 4.5-fold, compared to those who had normal blood pressure (SBP<160mmHg and DBP<95mmHg) in men and women.^19^ In a meta-analysis study in China, hypertensive patients had more than 5-fold greater risk of CVD than normotensives.^22^ Current methods of treatment for hypertension in China are non-drug treatment (including reasonable diet, weight control, and physical exercise) and drug treatment (including medicine and Chinese medicine).^23^ Generally, in rural areas in China only a few hypertension patients can receive treatments. In our study, there were 2,592 (5.2%) persons who had histories of hypertension; we regarded them as cases of hypertension. We found that both the ‘high-normal’ group and the ‘hypertension group’ showed a significant increase in risk of CVD mortality rate.

It has been suggested that diet has strong relationship with CVD.^24^ Sodium was an important dietary factor of CVD mortality. It was found that the geographic variations in salt consumption corresponded somewhat to the geographic distribution of both hypertension and CVD (including cerebral infarction, subarachnoid and intracerebral hemorrhage) in China.^25^ High salt intake increased the risk of both hypertension and CVD. The mechanism by which sodium may cause CVD was unclear. It was reported that high salt intake not only elevated blood pressure by expansion of blood volume, but also caused a direct damage to vessel walls through the acceleration of platelet aggregation.^24^ A restricted salt intake trial in Tianjin showed that blood pressure decreased substantially in both the hypertensive group and the normotensive group when following a low-sodium and high-potassium diet.^26^ In a case-control study in rural areas in China, it was reported that there was a significant increase in the risk of CVD in the population who had a preference for salty foods (estimated daily salt intake over 20g).^25^ In the present study, we found that reducing the degree of liking for salty foods significantly decreased CVD mortality rate. However, we found significantly inverse association between frequency of Chinese pickles intake and CVD mortality rate. We supposed that people who had high frequency of Chinese pickles intake were usually in a good nutrition state due to their good economic status. Another reason may be seasonal variation for Chinese pickles intake. Chinese pickles were usually taken in winter and early spring in the investigating areas, however the baseline survey was performed in summer or autumn. In the investigating areas, people are like to add salt to daily foods, such as vegetables. The consumption of salt in rural areas in Jiangxi Province was 19.2g per reference man per day,^27^ which was higher than the national average for rural areas in China (13.9g per reference man per day).^28^ This might be related to the fact that the health education about salt intake was not sufficient in the investigated areas.

Meat intake was an important protective dietary factor of CVD mortality in our study. In an ecological study of 600 geographic areas within Japan, intake of animal protein appeared to have a protective effect on CVD.^29^ Our study also found that the risk of CVD mortality among people in the group that had intake of meat once or twice per month was significantly decreased. This suggested that insufficiency of animal protein was a risk factor for CVD. According to The Dietary and Nutritional Status of Chinese Population in 1992, in all rural areas in Jiangxi Province, only 4.7% of the energy source was from animal food.^30^ The protein supplied by animals was only 9.9%,^30^ which was lower than that in rural areas in China nationwide (12.4%),^31^ and far lower than that in Japan in 1992 (53.0%).^32^ In Japan in the 1960s, low serum cholesterol caused by insufficiency of animal protein and by high intake of salt, as well as by strenuous labour-intensive work, elevated the risk of cerebral hemorrhage in rural areas.^33^ It is considered that the higher amount of death from cerebral hemorrhage (68.4%) in our study was caused by insufficient nutrition; paralleling conditions similar to those in Japan in the 1960s.^33^ No significant association was found between liking for fatty foods and risk of CVD mortality in our study.

Our data also suggested that ex-drinkers had a significantly increased risk for CVD mortality rate, compared with non-drinkers. Ex-drinkers often had health problems or doctor-diagnosed illnesses.^34^ Increased education regarding these drinking-related illnesses (coronary heart disease for instance) that might encourage patients to stop drinking.

We used food frequency questionnaire and food preference questionnaire in our study. Quantitative investigation of foods should also be conducted in order to give concrete information to be used for directing dietary habits in these areas.
